# Letter by Bohora Regarding Article, "Brugada-type Electrocardiographic Pattern Induced by Electrocution"

**Published:** 2009-03-15

**Authors:** Shomu Bohora

**Affiliations:** Consultant, Baroda Heart Institute and Research Centre, Vadodara, India

**Keywords:** Electrocution, Brugada syndrome, pericarditis

In response to the case report of Brugada-type Electrocardiographic Pattern Induced by Electrocution by R Rangaraj et al  [1], the electrocardiogram shows J point along with ST elevations in all the leads except lead III and ST depression in lead aVR. The ST segment is concave upwards in all leads along with an upright T wave except in lead V1. The characteristics features of the electrocardiogram have been classically described in pericarditis (with the exception of lead V1), which the authors should have considered as a differential diagnosis. Acute pericarditis due to electrocution also has not been described in literature prior. Hence electrocution leading to myo-pericarditis would have been a better way to probably describe the new entity.
